# Pulmonary mucoepidermoid carcinoma arising in a patient with Kartagener syndrome

**DOI:** 10.1186/s12890-020-1133-y

**Published:** 2020-04-16

**Authors:** Yoshiaki Nagai, Nobuyuki Koyama, Yuki Iwai, Hiroyoshi Tsubochi, Masahiro Hiruta, Yoshiko Mizushina, Shinichiro Koyama, Yuichi Ishikawa, Koichi Hagiwara

**Affiliations:** 1 Department of Respiratory Medicine, Jichi Medical University, Shimotsuke, Japan; 2 Department of Clinical Oncology, Tokyo Medical University Ibaraki Medical Center, Ibaraki, 300-0395 Japan; 3 Department of Respiratory Medicine, Tokyo Medical University Ibaraki Medical Center, Ibaraki, Japan; 4 Department of Respiratory Surgery, Jichi Medical University Saitama Medical Center, Saitama, Japan; 5 Department of Pathology, Jichi Medical University Saitama Medical Center, Saitama, Japan; 6 Department of Respiratory Medicine, Jichi Medical University Saitama Medical Center, Saitama, Japan; 7 Department of Pathology, Japanese Foundation for Cancer Research, Tokyo, Japan

**Keywords:** Kartagener syndrome, Mucoepidermoid carcinoma, Atelectasis, Female, Non-smoker

## Abstract

**Background:**

Kartagener syndrome, an autosomal recessive disorder with a triad of chronic sinusitis, bronchiectasis, and situs inversus, is characterized by recurrent respiratory tract infections and chronic inflammation of the lung. Information on comorbidities other than infections in patients with Kartagener syndrome is currently limited.

**Case presentation:**

A 39-year-old, non-smoking female was diagnosed with Kartagener syndrome and admitted to Saitama Medical Center, Jichi Medical University, Japan. Computed tomography revealed an endobronchial massive shadow at the ostial site of the right upper lobe bronchus with atelectasis of the right upper lobe. The mass was surgically resected and pathologically diagnosed as mucoepidermoid carcinoma. The lesion had no vascular invasions and no metastases to the lungs or lymph nodes. The surgical margin was negative for carcinoma. Following surgery, the patient has been in good condition.

**Conclusions:**

The present case showed different clinicopathological characteristics from those previously reported in cases of Kartagener syndrome complicated by carcinoma. To the best of our knowledge, this is the first reported case of a young, non-smoking female with comorbid Kartagener syndrome and pulmonary mucoepidermoid carcinoma. This case report may provide a new perspective on the complications of Kartagener syndrome.

## Background

Kartagener syndrome is an autosomal recessive disorder with a triad of chronic sinusitis, bronchiectasis, and situs inversus [1]. Although the estimated incidence is low (1/30000–1/60000 births) [2], fewer patients reach a definitive diagnosis due to an inadequate diagnostic strategy. This disease is characterized by recurrent respiratory tract infections and chronic inflammation of the lung due to congenital ciliary dyskinesia and impaired mucociliary clearance, associated with bronchiectasis and chronic respiratory failure [3, 4]. Radical treatment for Kartagener syndrome has not been established, and therapeutic strategies have been primarily focused on the control of infection. Currently, information on comorbidities other than infections in patients with Kartagener syndrome is limited.

This article presents a young, non-smoking female with Kartagener syndrome complicated by lung cancer. Histologically, the lung cancer arising from the right upper lobe bronchus was a mucoepidermoid carcinoma, a rare type of neoplasm accounting for 0.1–0.2% of all lung tumors [5]. Pulmonary mucoepidermoid carcinoma is characterized by low-grade malignancy, development from the salivary glands of the proximal tracheobronchial tree, and the t (11;19) chromosomal translocation generating a *CREB-regulated transcription coactivator 1-mastermind-like 2* (*CRTC1-MAML2)* fusion oncogene [6, 7]. The association between Kartagener syndrome and lung cancer has rarely been reported. Previous reports presented six patients with Kartagener syndrome and lung cancer [8–13]. Of those, four patients had squamous cell carcinoma, one patient had small cell carcinoma, and one patient had bronchogenic carcinoma. All patients were male, aged > 50 years, and heavy smokers.

Thus far, there are no reports showing the history of mucoepidermoid carcinoma. The patient presented in this report had different clinicopathological characteristics from those previously reported, and may provide a novel perspective on Kartagener syndrome.

## Case presentation

A 39-year-old female non-smoker was admitted to Saitama Medical Center, Jichi Medical University, Japan and received continuous care for Kartagener syndrome. The patient was previously admitted to a hospital for productive cough, yellow sputum, and nasal congestion. Chest computed tomography showed bronchiectasis and nodular shadows in both lungs, in addition to situs inversus.

The family history of the patient included nasal disorder (eldest brother) and situs inversus (second elder brother expired at 24 days old). The parents of the patient were cousins. Biopsy of the patient’s mucosa in the nasal cavity was performed in the otorhinolaryngology department. Based on the pathological findings, family history, and a triad of chronic sinusitis, bronchiectasis, and situs inversus, the physicians reached the diagnosis of Kartagener syndrome. Moreover, the patient had been diagnosed with chronic obstructive pulmonary disease, and had received treatment with macrolide antibiotics, inhaled corticosteroids/long-acting β2 agonists, and tiotropium.

**Fig. 1:**
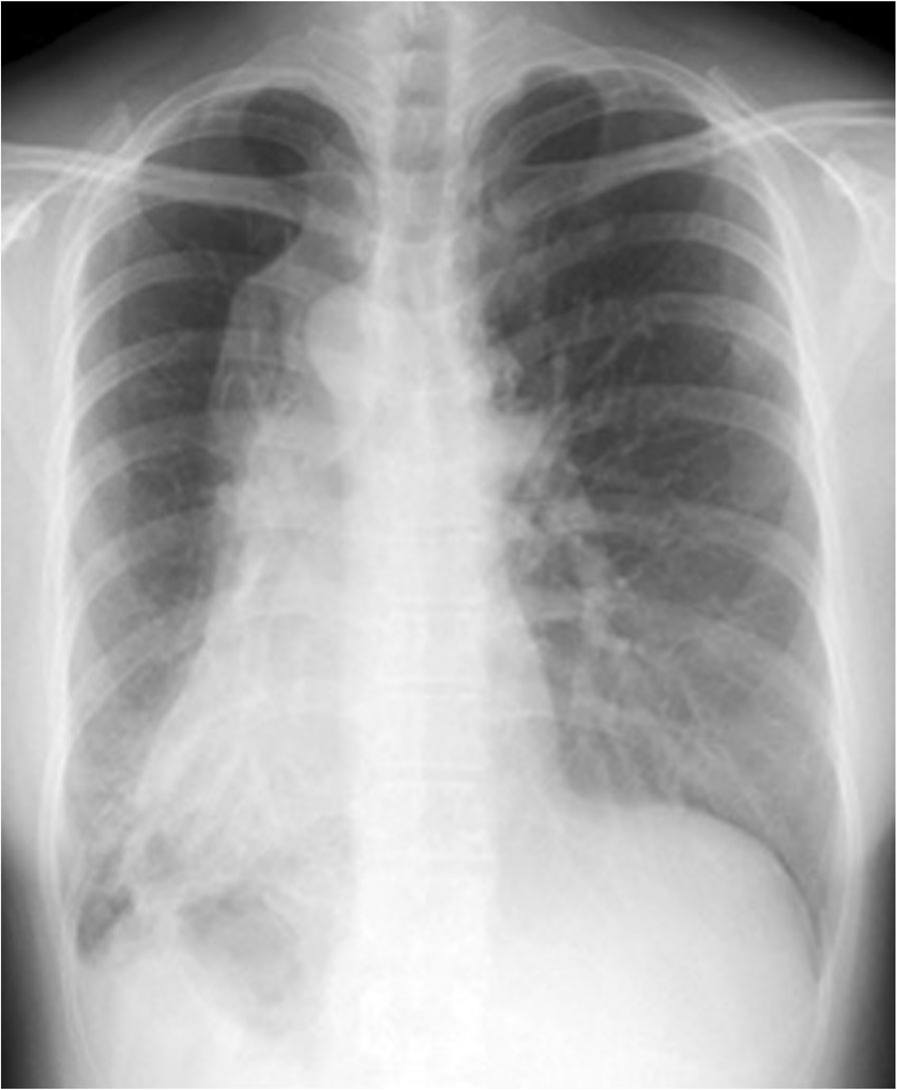
Chest X-ray examination performed on admission. Chest X-ray image showing an infiltrative shadow in the right upper lung field suggesting atelectasis of the right upper lobe and tram lines with nodular shadows in both lung fields

**Fig. 2:**
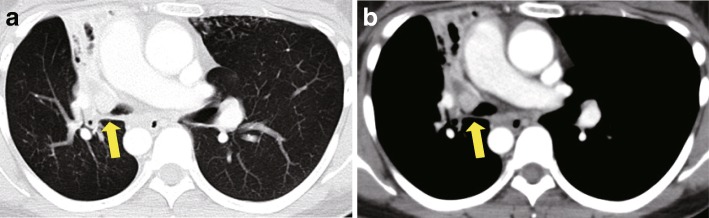
Chest computed tomography images. Chest computed tomography scan revealing an endobronchial massive shadow at the ostial site of the right upper lobe bronchus associated with atelectasis of the right upper lobe (indicated by arrows)

**Fig. 3:**
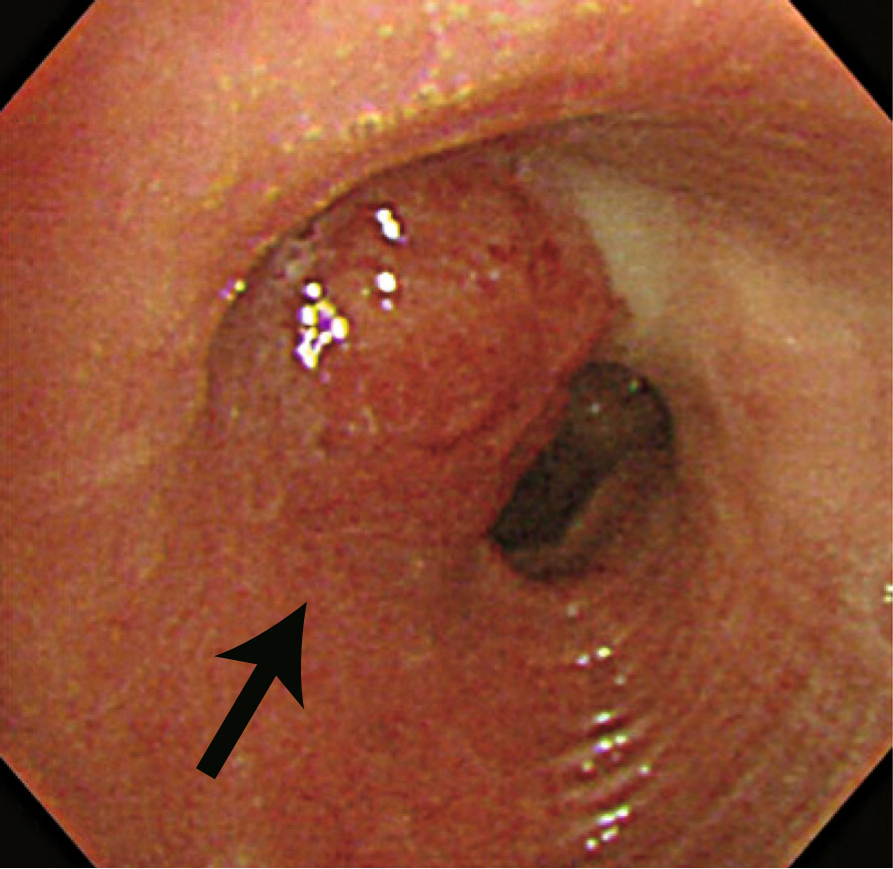
Bronchoscopic images. Bronchoscopy identified a mass in the upper lobular bronchus with obstruction of its peripheral airway (indicated by an arrow)

**Fig. 4:**
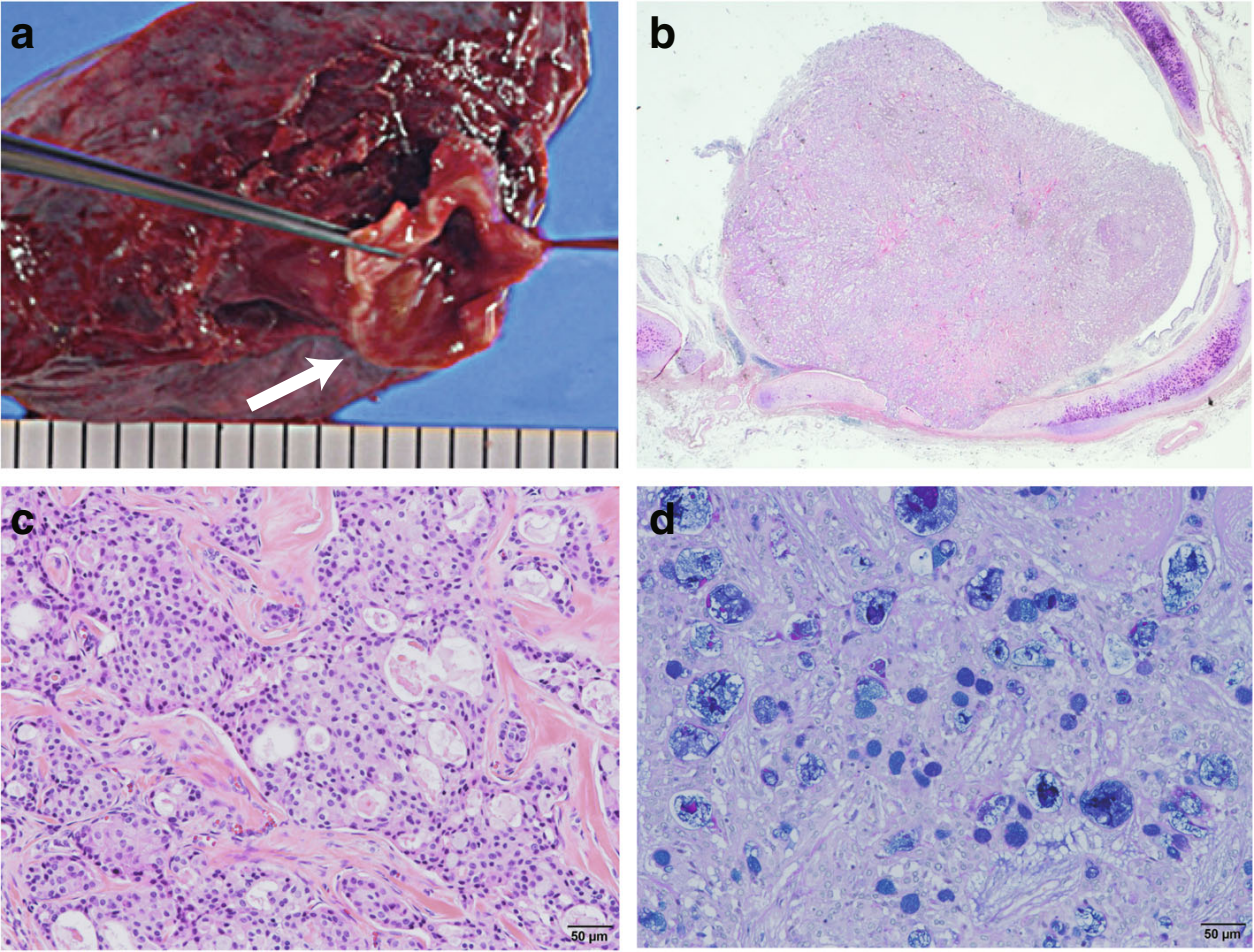
Pathological findings of the resected tumor. **a:** An opalescent tumor at the right bronchus was identified at the macroscopic scale (indicated by an arrow). **b:** A polypoid tumor successively arising from bronchial glands infiltrated into the bronchial cartilage without extrabronchial spread (hematoxylin and eosin staining, 5×). **c, d:** Mucoepidermoid carcinoma with mixed mucus-producing and squamous epithelia proliferated in a glandular-forming manner (**c** hematoxylin and eosin staining, 100×; **d** Alcian blue-periodic acid-Schiff staining, 100×)

Chest X-ray examination performed in our hospital showed an infiltrative shadow in the right upper lung field suggesting atelectasis of the right upper lobe and tram lines with nodular shadows in both lung fields (Fig. 1). The computed tomography images revealed an endobronchial massive shadow at the ostial site of the right upper lobe bronchus and atelectasis of the right upper lobe (Fig. 2). Bronchoscopic examination identified an obstructing mass at the orifice of the upper lobular bronchus (Fig. 3). Although biopsy failed to achieve a definitive diagnosis, the mass was surgically resected because neoplastic disease was suspected and airway obstruction required surgical intervention. The parts of the right lung chronically infected with *Pseudomonas aeruginosa* were concomitantly resected. Surgery included the resection of the right upper lobe and the right lower lobe with a preserved right S6 region through anastomosis between the right B6 and the right main bronchus due to situs inversus. In the resected lung, an opalescent tumor was observed at the right bronchus (Fig. 4a). Microscopically, the tissue specimen revealed a polypoid tumor infiltrating into the bronchial cartilage at low-magnification (Fig. 4b). Histopathological analysis of the tumor provided evidence of mucoepidermoid carcinoma (Figs. 4c, d). Common driver oncogenes and biomarkers in the tumor were negative for mutations in the gene encoding *epidermal growth factor receptor* (*EGFR*)/*v-raf murine sarcoma viral oncogene homolog B1* (*BRAF*) or rearrangements in the *anaplastic lymphoma kinase* (*ALK*)/*c-ros oncogene 1* (*ROS1*)/*rearranged during transfection proto-oncogene* (*RET*) genes, or the expression of programmed death ligand (PD-L1). The *CRTC1-MAML2* gene fusion was not analyzed in this case. The carcinoma arising from a bronchial gland was 1.6 cm × 1.4 cm in size and invaded into the bronchial cartilage; however, it did not extend outside the bronchus. There was no vascular invasions or metastases to the lungs or lymph nodes. The surgical margin was negative for carcinoma. The right lower lobe showed histopathological findings consistent with Kartagener syndrome (e.g., included alveolar wall thickening, organizing pneumonia with inflammatory cell invasion, bronchiectasis, and enlarged alveolar spaces). Following surgery, the patient has been free of relapse with a decreased incidence of pneumonia.

## Discussion and conclusions

This report presents a case of Kartagener syndrome in a young, non-smoking female complicated by mucoepidermoid carcinoma, a rare histological subtype of lung cancer. To the best of our knowledge, this is the first reported case of a young, non-smoking female with comorbid Kartagener syndrome and lung cancer. The onset of Kartagener syndrome, an autosomal recessive disease, is not associated with age, sex, or history of smoking. In contrast, lung cancer occurs more frequently in the elderly, males, and smokers. Papi et al. reported that chronic inflammation due to emphysema and smoking increased the risk of pulmonary squamous cell carcinoma [14]. In previous reports, most patients with Kartagener syndrome who developed lung cancer were smoking males [8–13]. However, mucoepidermoid carcinoma commonly originating from salivary glands exhibits different clinical features from other lung cancers. In the present case, the tumor did not harbor driver oncogenes including, *EGFR* mutations, which are frequently found in non-smoking females with non-small cell lung cancer. Previous case reports have suggested the possible role of congenital lung malformation in the onset of mucoepidermoid carcinoma [15, 16]. Thus, the histological subtype of mucoepidermoid carcinoma may be etiologically associated with Kartagener syndrome, congenital ciliary dyskinesia, although the biological mechanism remains unknown.

Kartagener syndrome associated with recurrent respiratory tract infections and bronchiectasis is pathophysiologically characterized by chronic inflammation of the lungs. Some researchers reviewed the association of chronic inflammation with tumorigenesis [17, 18]. Chung et al. reported an increased risk of lung cancer in patients with bronchiectasis, one of the clinical phenotypes of Kartagener syndrome [19]. In this context, the pathophysiological process of Kartagener syndrome may have an impact on the onset of mucoepidermoid carcinoma. However, Kim et al. showed inverse comorbidity relationships between lung cancer and chronic inflammation caused by bronchiectasis [20, 21]. Furthermore, Mak et al. reported that patients with bronchiectasis have high levels of transforming growth factor β1 in the serum, a potential protective factor against carcinogenesis [22]. Li et al. also reported low incidence of comorbid lung cancer in patients with cystic fibrosis characterized by bronchiectasis [23]. The role of the pathophysiological process of Kartagener syndrome in the risk of developing carcinoma is controversial.

In the context of the pathogenesis of lung cancer in patients with Kartagener syndrome, the different clinicopathological characteristics of the patient in the present report from those reported in previous cases suggest that different mechanisms may develop malignancy of the lung in patients with Kartagener syndrome [13]. A case series analysis is warranted to determine whether mucoepidermoid carcinoma is causally associated with Kartagener syndrome.

In conclusion, this article presents the first reported case of pulmonary mucoepidermoid carcinoma developed in a patient with unusual characteristics for lung neoplasms such as young age, female sex, no history of smoking, and presence of Kartagener syndrome. These findings emphasize the need for caution following the onset of malignancy as a complication in patients with Kartagener syndrome and unusual clinicopathological characteristics.

## Data Availability

All data generated during this study are included in this published article.

## Abbreviations

ALK: Anaplastic lymphoma kinase
BRAF: V-raf murine sarcoma viral oncogene homolog B1
CRTC1-MAML2: CREB-regulated transcription coactivator 1-mastermind-like 2
EGFR: Epidermal growth factor receptor
PD-L1: Programmed death ligand
RET: Rearranged during transfection proto-oncogene
ROS1: C-ros oncogene 1
